# Priority regions for research on dryland cereals and legumes

**DOI:** 10.12688/f1000research.8657.2

**Published:** 2016-07-07

**Authors:** Glenn Hyman, Elizabeth Barona, Chandrashekhar Biradar, Edward Guevara, John Dixon, Steve Beebe, Silvia Elena Castano, Tunrayo Alabi, Murali Krishna Gumma, Shoba Sivasankar, Ovidio Rivera, Herlin Espinosa, Jorge Cardona

**Affiliations:** 1 International Center for Tropical Agriculture (CIAT), Cali, Colombia; 2 Independent Researcher, Miami, USA; 3 International Center for Agricultural Research in the Dry Areas (ICARDA), Beirut, Lebanon; 4 Australian Centre for International Agricultural Research, Canberra, Australia; 5 International Institute of Tropical Agriculture (IITA), Ibadan, Nigeria; 6 International Crops Research Institute for the Semi-Arid Tropics (ICRISAT), Patancheru, India

**Keywords:** Grain legumes, dryland cereals, farming systems, priority setting, geographic priorities

## Abstract

Dryland cereals and legumes  are important crops in farming systems across the world.  Yet they are frequently neglected among the priorities for international agricultural research and development, often due to lack of information on their magnitude and extent. Given what we know about the global distribution of dryland cereals and legumes, what regions should be high priority for research and development to improve livelihoods and food security? This research evaluated the geographic dimensions of these crops and the farming systems where they are found worldwide. The study employed geographic information science and data to assess the key farming systems and regions for these crops. Dryland cereal and legume crops should be given high priority in 18 farming systems worldwide, where their cultivated area comprises more than 160 million ha. These regions include the dryer areas of South Asia, West and East Africa, the Middle East and North Africa, Central America and other parts of Asia. These regions are prone to drought and heat stress, have limiting soil constraints, make up half of the global population and account for 60 percent of the global poor and malnourished. The dryland cereal and legume crops and farming systems merit more research and development attention to improve productivity and address development problems. This project developed an open access dataset and information resource that provides the basis for future analysis of the geographic dimensions of dryland cereals and legumes.

## Introduction

International agricultural research and development programs usually consider the geographic dimensions of crop improvement and farming systems in their efforts to prioritize activities ( Hyman *et al.*, 2013). Where is the crop grown globally and what are the key obstacles to crop production? Does the research apply to places where benefits can reach a large number of people? How can resources be allocated to achieve efficiencies? But answering these questions requires integration of socioeconomic and biophysical data and the integration of wide-ranging data and information resources. Often data exists for national jurisdictions, but it needs to be evaluated by agroecology or farming system. The task is even more difficult for crops such as dryland cereals and grain legumes because – when compared to the major staple crops – these crops are often embedded in complex crop-livestock systems and less information is available. The Dryland Cereals and Legumes Agri-Food System research program of the CGIAR (the program is hereafter referred to as DCL) requested an analysis of the principal commodities of their proposed program and the farming systems in which they are found. The 12 priority crops of these dryland systems are chickpea, common bean, cowpea, faba bean, groundnut, lentil, pigeon pea, soybean, barley, pearl millet, small millet and sorghum ( DCL, 2015). The research presented in this paper shows the development of spatial and statistical data intended to support geographic priority setting for the global DCL research program. In order to develop the analysis, this research builds on a global classification of farming systems, on maps of the spatial distribution of all 12 DCL crop commodities, on socioeconomic data on population, poverty, malnutrition, on market access, and on soil and climatic data. The analysis identifies where these crops occur in the context of constraints and opportunities for their development. How can DCL technologies be geographically targeted for achieving the objective of reducing poverty and malnutrition in dryland systems? The present analysis is based on a diverse array of geographic information, and includes new assessments of poverty, drought, heat and other information related to crop improvement and management. In this way, the study examines the spatial extents of key constraints to DCL crop production, using the most recent spatial data available. The analysis and resulting database provides the first global farming systems information resource for specifically evaluating priorities for DCL crop improvement and management. Tabular data from this analysis is open access and has been published in a data repository ( Barona *et al.*, 2016a). The geospatial data used this study can be accessed through a new online digital atlas for dryland cereals and grain legumes (see http://www.eatlasdcl.cgiar.org/).

## Methodology

This study builds on previous work (including Dixon *et al.*, 2001 and Hyman *et al.*, 2008), but with a focus on the 12 principal commodities and farming systems of DCL. The main framework for the study is John Dixon’s farming systems framework, a global delineation and resulting map of the major farming systems of the developing world ( Dixon *et al.*, 2001). Dixon’s schema is built on 15 biophysical and socioeconomic spatial data layers available in the year 2000 and consultations with hundreds of regional and global agricultural experts using a modified Delhi technique. In a participatory process, 72 global farming systems in six developing regions were geographically delineated and their characteristics described. The present study uses a subset of 63 of those farming systems together with new spatial data on biophysical and socioeconomic conditions to characterize the extents of DCL commodities. Using spatial overlay, biophysical and socioeconomic information are organized according to the 63 Dixon farming systems.

A key advantage of this research was that instead of analyzing crop information by country, subnational estimates of crop distribution are generated based on pixel level data ( Hyman *et al.*, 2008). Then, using spatial overlay, we organize that data by country (250 in total), by farming system (63 types) and by combinations of countries and farming systems (544 combinations). Other data is also organized according to farming system and country – including information on drought, temperature dynamics with climate change, soil conditions, population and poverty. Readers should consult our previous publication and available data for further details on the data and methodology ( Barona *et al.*, 2016a and Barona *et al.*, 2016b; Hyman *et al.*, 2008; Hyman *et al.*, 2015).

### Data sources

Spatial information on biophysical and socioeconomic conditions was acquired, with the objective of obtaining the most recent and spatially detailed information related to dryland cereals and legume and the agricultural systems where they are found. The present study upgrades our previous work because we are using data that was not available before, especially the 2005 spatial distribution of crop area, production and yield ( You & Wood, 2006; You *et al.*, 2014a; You *et al.*, 2014b). The previous study used crop distribution data from the year 2000, while the work we describe here uses 2005 crop distribution data. Our previous dataset only included 7 DCL commodities, in contrast to all 12 of the DCL commodities used here. These new data also benefited from improved spatial resolution and modeling procedures. This study used the most recent available data on global livestock and human population. The source of the year 2010 human population data was the gridded population of the world project ( CIESIN, 2014). Livestock population was taken from the Gridded Livestock of the World (GLW) database at 5 km spatial resolution, with the year 2005 as the reference year ( Robinson *et al.*, 2014).

Several datasets gave us information on abiotic constraints to crop production that are important for the DCL commodities. The dataset includes indicators of drought based on maps of drought probability and the “failed seasons” concept. By simulating rainfall for defined crop water requirements, the probability of a growing season failing to produce a successful harvest indicates drought risk for every pixel across the world ( Hyman *et al.*, 2008; Jones & Thornton, 2000; Jones *et al.*, 2002). The drought probability is multiplied by total crop area to derive the potential drought impact index (PDII). Furthermore, the needed heat tolerance for DCL crops was indicated by estimates of expected temperature change between the current temperature and 2050 temperatures ( Hijmans *et al.*, 2005, Ramirez & Jarvis, 2008). These predicted changes are based on global circulation models (GCM) under the A1B scenario, assuming rapid economic growth with emissions peaking around 2050. The study used maps of soil constraints based on the fertility capability classification ( Sanchez *et al.*, 2003). These constraints included soil acidity, length of the dry season, waterlogging, low nutrient availability and salinity – all constraints identified by DCL crop experts as important obstacles to overcome ( DCL, 2015). Finally, the length of the growing period indicates seasonal constraints on crops that may be relevant for the breeding objectives of DCL crops ( Fischer, 2009). 

Detailed geographic information on population is not usually available until at least five years after the dates of censuses and surveys. Our analysis includes estimates of the total population for the year 2010, as well as total, rural and urban population for 2005 ( CIESIN *et al.*, 2005; CIESIN, 2014). We included 2005 population data in our analysis because the CIESIN (2014) population dataset does not yet include urban and rural data for 2010. The analysis draws on estimates of the number of people living on less than $1 and $2 per day for 10 km pixel areas, based on estimates derived from combined poverty maps and survey data for the entire world, with a base year of 2005. This global poverty data set is not available in the public domain, but interested users of poverty data should consult the HarvestChoice website to learn more about this and other poverty mapping initiatives (Stanley Wood, personal communication). Nutrition indicators include the absolute number and proportional numbers of children under five years old that are two standard deviations below the median of weight for age (underweight) and height for age (stunting), according to international standards ( CIESIN, 2005; FAO, 2007).

### Spatial analysis

Spatial overlay was used to organize the data into spatial units according to farming system and combinations of farming systems and country. All spatial data was converted to the Robinson equal area projection at 10 km spatial resolution before processing commenced. We used the zonal statistics tools in ArcInfo Workstation 10.0 and ArcGIS Desktop 10.1 software. The analysis digitally overlays Dixon’s farming systems map and a global map of country boundaries on the socioeconomic and biophysical map data described above. The result of the overlay procedure is a set of database files (dBase format) organized by farming system region and combination of farming system region and country. The database files were then converted to 40 spreadsheet files in Excel format ( Barona *et al.*, 2016b). An additional analysis was made of the pixels where more than one DCL crop occurred within the pixel. For each crop, if the area value in the pixel was higher than the mean for all pixels of that crop, it was considered to be of a sufficient density to map these crop combinations. By selecting only those pixels above the mean, we excluded those areas that may have a small concentration of the crop. The creation of the tables was facilitated using scripts written in Arc Macro Language (AML) to facilitate updates as more recent or better data becomes available (scripts and data available from Barona *et al.*, 2016b).

### Determining priority regions

The study used a mix of criteria for determining priority regions for research and development in DCL farming systems. A modified “natural breaks” approach was our primary consideration in selecting priority farming system regions. Building on the determination of classes for choropleth mapping, the approach visually inspects the data to find where farming system regions group together according to their levels of DCL crop area ( Smith, 1986). We also considered whether a single crop dominated a farming system region in areas with large farms and well-developed agro-industries. Some farming systems that have relatively small DCL crop areas could be included if they were similar to regions that have large areas, something that often occurs with farming systems in different regions of the world.

Another criteria was whether these farming systems overlapped with DCL program target countries, as established in the DCL pre-proposal ( DCL, 2015). A key criteria for program target countries is that they fall within dryland regions, defined as areas with an aridity index between 0.03 and 0.65 ( Zomer *et al.*, 2008). A dryland regions map and a description of how it was made can be accessed on the online DCL Atlas. Beyond this consideration, the DCL program’s target country analysis considered crop area and production, people living in poverty, childhood malnutrition, land degradation and other considerations – all with national level data. The reference map in the online atlas contains the map of target countries of the DCL program.

The approach we describe above leaves open the possibility to select different criteria and to expand or contract the number of farming systems that would be targeted for a research and development program. The publication of replication data found in the online atlas and in the Dataverse repository enable future iterations of the analysis according to any adjustments that the program may want to make ( Barona *et al.*, 2016a).

## Results

**Figure 1.**
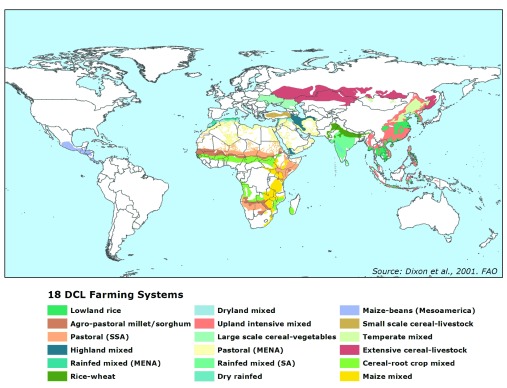
18 priority dryland cereal and legume priority farming systems worldwide.

The DCL crops are concentrated in 18 farming systems where more than 160 million ha of these crops are cultivated, where more than 60% of the world’s poor live and where the DCL commodity programs have selected target countries based on their fit with dryland systems ( Figure 1; Table 1 & Table 7; DCL, 2015; DESA, 2009). We selected these farming systems if the farming system had at least six million ha of combined DCL crops. However, we excluded three Latin American and Caribbean (LAC) farming systems that met this threshold because these systems were overly dominated by soybean production in regions with typically large farms. These excluded systems were *temperate mixed* (Pampas), *cereal-livestock* (Campos) and *extensive mixed* (Cerrados_Llanos).

**Table 1.** DCL crop area in ‘000’s of hectares in 18 priority farming systems worldwide.

FARMING SYSTEMS	REGION	BARL	BEAN	CHKP	COWP	GRDN	LENT	PMIL	PSML	PPEA	SORG	SOYB	FABAB	TOTAL
**Cereal-root** **crop mixed**	SSA	26	573	32	2,983	2,949	1	4,649	128	78	9,594	295	19	**21,328**
**Maize mixed**	SSA	81	2,175	107	387	977	7	655	432	431	1,976	309	70	**7,607**
**Agro-pastoral** **millet/sorghum**	SSA	2	169	1	3,489	1,751	0	7,551	0	8	5,596	108	15	**18,691**
**Pastoral**	SSA	65	77	21	2,070	725	7	4,798	9	0	2,955	14	66	**10,808**
**Rice-wheat**	SA	461	1,575	1,966	0	277	977	4,012	144	543	966	362	0	**11,283**
**Rainfed mixed**	SA	161	3,951	4,062	4	4,014	595	2,628	1,697	2,149	4,226	7,276	0	**30,763**
**Dry rainfed**	SA	0	496	1,030	0	1,168	0	1,148	68	735	3,829	210	0	**8,685**
**Highland** **mixed**	MENA	1,704	83	524	0	1	189	1	67	0	291	75	28	**2,961**
**Rainfed mixed**	MENA	1,197	22	94	0	26	69	5	2	0	17	1	156	**1,589**
**Dryland mixed**	MENA	3,486	6	126	0	7	125	0	11	0	10	7	62	**3,841**
**Pastoral**	MENA	737	18	40	0	13	37	0	19	0	120	4	11	**1,001**
**Maize-beans** **(Mesoamerica)**	LAC	277	783	30	0	23	8	0	0	1	597	15	17	**1,750**
**Large scale** **cereal-** **vegetable**	EECA	5,927	44	2	0	0	1	0	309	0	30	634	1	**6,948**
**Small scale** **cereal-** **livestock**	EECA	2,057	59	235	0	6	181	0	2	0	0	0	9	**2,550**
**Extensive** **cereal-** **livestock**	EECA	8,322	5	12	0	0	6	0	535	0	22	250	6	**9,161**
**Lowland rice**	EAP	408	2,436	1	39	2,805	16	53	28	18	91	2,696	187	**8,778**
**Upland** **intensive** **mixed**	EAP	154	1,167	86	65	1,629	20	522	25	436	170	3,336	258	**7,869**
**Temperate** **mixed**	EAP	75	259	1	0	1,202	16	209	3	0	264	4,178	333	**6,539**
**TOTAL**		**25,141**	**13,899**	**8,371**	**9,039**	**17,576**	**2,257**	**26,233**	**3,477**	**4,399**	**30,754**	**19,770**	**1,236**	**162,152**

Priority DCL farming systems were included from Latin America and the Middle East and North Africa (MENA) regions that did not meet the six million hectare threshold described above. The *maize-beans* farming system in Mesoamerica was added because it is very similar to *maize mixed* system in sub-Saharan Africa. The *rainfed mixed* and *pastoral* farming systems in the MENA region were added because they are similar to farming systems in sub-Saharan Africa ( *pastoral*) and in South Asia ( *rainfed mixed*). Two other farming systems – *dry rainfed* and *highland mixed* – are included on the basis of traditional importance in the dryland MENA region.

### Production, area and yield

Three farming systems in South Asia – *rainfed mixed, rice-wheat* and *dry rainfed* – make up about one third of the 162 million ha of DCL crops in the 18 priority farming systems ( Table 1). The *rainfed mixed* system makes up 20% of the DCL crop area in these priority farming systems, accounting for more than 30 million ha of DCL crops. A second important region is Sub-Saharan Africa, where the *cereal-root crop mixed* system accounts for 21.3 million ha, the *agro-pastoral millet sorghum* system accounts for 18.6 million ha, the *pastoral* system accounts for 10.8 million ha and the *maize mixed* system has 7.6 million ha. In Eastern Europe and Central Asia more than 15 million ha are cultivated, with barley figuring prominently. In East Asia over 22 million ha are cultivated, with groundnut and soybean as the predominant crops. The overall DCL crop area in the Middle East and North Africa is the lowest among the 18 priority regions suggested above.

In some cases DCL crops make up a large proportion of the total cultivated area in these farming systems, but their overall area may be relatively small when they are found in systems with large areas of maize, wheat and rice ( Table 2). Three cereals (barley, pearl millet and sorghum) and two legumes (soybean and cowpea) play key roles in several farming systems where they make up more than 10% of all cultivated crop area within the system. The legumes are multi-purpose, contributing soil fertility, family nutrition, fodder and cash sales. Seven relatively cooler farming systems have more than 13 percent of their crop area in barley – four of which are in the Middle East and North Africa and three in Eastern Europe and Central Asia region. Pearl millet is an important component in the higher temperature *pastoral*, *agro-pastoral millet*- *sorghum* and *cereal-root crop mixed farming* systems, making up 35%, 32% and 11% of the total cultivated area respectively. In the *pastoral* and *agro-pastoral millet*- *sorghum* systems pearl millet is a major food crop, whereas in the somewhat higher rainfall *cereal-root crop mixed* system maize, sorghum and cassava are the major food crops. In three African systems and one South Asian system, sorghum makes up more than 20% of the total cultivated area, although being pushed back by drought tolerant maize. In five of these 18 farming systems groundnuts make up between five and eight percent of the total cultivated area. The remaining crops – bean, chickpea, lentil, small millet and pigeon pea – have a smaller overall agricultural footprint.

**Table 2.** Proportion of the total cultivated area by farming system. In some cases DCL crops make up a large proportion of the total cultivated area in these farming systems, but their overall area may be relatively low when they are found in systems with large areas in maize, wheat and rice.

FARMING SYSTEMS	REGION	BARL	BEAN	CHKP	COWP	GRDN	LENT	PMIL	PSML	PPEA	SORG	SOYB	FABAB
**Cereal-root crop** **mixed**	SSA	0.00064	0.01421	0.00080	0.07398	0.07316	0.00004	0.11533	0.00317	0.00193	0.23797	0.00733	0.00047
**Maize mixed**	SSA	0.00341	0.09188	0.00453	0.01636	0.04129	0.00031	0.02766	0.01824	0.01821	0.08346	0.01306	0.00294
**Agro-pastoral** **millet/sorghum**	SSA	0.00010	0.00733	0.00005	0.15099	0.07581	0.00000	0.32683	0.00000	0.00034	0.24220	0.00467	0.00066
**Pastoral**	SSA	0.00474	0.00563	0.00157	0.15123	0.05296	0.00048	0.35049	0.00066	0.00001	0.21586	0.00106	0.00482
**Rice-wheat**	SA	0.00549	0.01876	0.02342	0.00000	0.00330	0.01163	0.04780	0.00171	0.00647	0.01150	0.00431	0.00000
**Rainfed mixed**	SA	0.00214	0.05251	0.05398	0.00006	0.05335	0.00791	0.03492	0.02256	0.02856	0.05616	0.09671	0.00000
**Dry rainfed**	SA	0.00000	0.03485	0.07233	0.00000	0.08198	0.00000	0.08058	0.00477	0.05162	0.26876	0.01475	0.00000
**Highland mixed**	MENA	0.14739	0.00714	0.04530	0.00000	0.00007	0.01634	0.00008	0.00575	0.00000	0.02519	0.00649	0.00239
**Rainfed mixed**	MENA	0.15464	0.00280	0.01208	0.00002	0.00335	0.00895	0.00062	0.00021	0.00000	0.00216	0.00017	0.02018
**Dryland mixed**	MENA	0.27190	0.00049	0.00982	0.00002	0.00057	0.00978	0.00002	0.00082	0.00000	0.00081	0.00052	0.00481
**Pastoral**	MENA	0.13266	0.00331	0.00726	0.00004	0.00242	0.00668	0.00003	0.00348	0.00000	0.02154	0.00066	0.00202
**Maize-beans** **(Mesoamerica)**	LAC	0.02830	0.08007	0.00310	0.00000	0.00240	0.00079	0.00000	0.00000	0.00007	0.06104	0.00150	0.00169
**Large scale** **cereal-vegetable**	EECA	0.18685	0.00139	0.00007	0.00000	0.00000	0.00003	0.00000	0.00974	0.00000	0.00095	0.01998	0.00003
**Small scale** **cereal-livestock**	EECA	0.22776	0.00654	0.02603	0.00000	0.00070	0.02009	0.00000	0.00020	0.00000	0.00000	0.00005	0.00101
**Extensive** **cereal-livestock**	EECA	0.16105	0.00010	0.00024	0.00000	0.00000	0.00012	0.00000	0.01036	0.00000	0.00043	0.00484	0.00012
**Lowland rice**	EAP	0.00366	0.02182	0.00000	0.00035	0.02513	0.00014	0.00048	0.00025	0.00016	0.00082	0.02415	0.00167
**Upland** **intensive mixed**	EAP	0.00226	0.01719	0.00126	0.00096	0.02400	0.00030	0.00769	0.00036	0.00643	0.00250	0.04914	0.00379
**Temperate** **mixed**	EAP	0.00200	0.00692	0.00001	0.00000	0.03213	0.00043	0.00559	0.00008	0.00000	0.00706	0.11167	0.00889

Yields vary across the 18 farming systems and by DCL commodity ( Table 3). A very general pattern is that yields are lowest in sub-Saharan Africa. They are somewhat higher in South Asia and even more so in Eastern Europe and Central Asia. Finally they are highest in the East Asian countries. These differences are related to many different factors, including population density, access to agricultural services and markets, biotic and abiotic constraints, technology levels, management practices and others.

**Table 3.** Dryland cereal and legume crop yield in 18 priority farming systems worldwide.

FARMING SYSTEMS	REGION	BARLEY (YIELD kg/ha)	BEAN (YIELD kg/ha)	CHICKPEA (YIELD kg/ha)	COWPEA (YIELD kg/ha)	GROUNDNUT (YIELD kg/ha)	LENTIL (YIELD kg/ha)	PEARL- MILLET (YIELD kg/ha)	SMALL- MILLET (YIELD kg/ha)	PIGEONPEA (YIELD kg/ha)	SORGHUM (YIELD kg/ha)	SOYBEAN (YIELD kg/ha)	FABA BEAN (YIELD kg/ha)
**Cereal-root** **crop mixed**	SSA	1,184	477	655	638	1,177	759	1,150	751	622	1,011	849	1,617
**Maize mixed**	SSA	1,837	525	465	564	724	738	551	1,436	671	859	1,155	1,071
**Agro-pastoral** **millet/sorghum**	SSA	3,107	477	576	365	830	0	736	0	658	769	565	1,771
**Pastoral**	SSA	1,254	562	820	217	714	894	471	1,585	604	611	531	1,144
**Rice-wheat**	SA	2,087	491	735	0	822	830	1,171	1,046	939	659	1,411	0
**Rainfed mixed**	SA	1,559	339	842	1,029	1,063	512	832	850	708	910	1,003	0
**Dry rainfed**	SA	0	212	895	0	716	0	704	809	495	687	785	0
**Highland** **mixed**	MENA	1,418	1,912	505	667	2,416	490	1,776	694	0	805	2,383	646
**Rainfed mixed**	MENA	915	793	659	722	2,456	906	1,874	823	0	804	1,461	560
**Dryland mixed**	MENA	933	1,554	644	759	2,536	942	1,586	1,046	0	1,208	2,313	707
**Pastoral**	MENA	1,416	2,529	717	2,302	2,716	689	1,412	721	0	2,019	3,188	2,138
**Maize-beans** **(Mesoamerica)**	LAC	2,866	680	1,535	0	1,459	885	0	0	424	4,571	2,487	706
**Large scale** **cereal-** **vegetable**	EECA	2,240	1,375	2,051	0	0	1,311	0	1,207	0	1,958	1,373	1,135
**Small scale** **cereal-** **livestock**	EECA	2,705	1,534	1,036	0	3,195	1,312	0	1,949	0	0	4,131	1,870
**Extensive** **cereal-** **livestock**	EECA	1,619	1,679	967	0	1,607	971	2,362	1,039	0	1,421	1,149	1,192
**Lowland rice**	EAP	4,253	1,074	4,604	1,000	2,964	2,423	1,855	1,027	1,013	3,756	1,764	1,640
**Upland** **intensive** **mixed**	EAP	3,383	1,008	1,234	969	2,150	1,616	1,775	934	1,041	3,555	1,474	1,640
**Temperate** **mixed**	EAP	4,320	1,423	3,555	0	3,380	1,783	2,384	1,040	0	4,698	1,822	1,640

### Livestock and DCL farming systems

Livestock is an important component of the DCL research program and all the farming systems where DCL crops are concentrated. The DCL crops are considered multi-purpose crops because they are used for many purposes including food, feed and fodder – as well as ecosystem services. Soybean and barley are perhaps the most important for livestock, with much of their production going towards animal fodder ( Hartman *et al.*, 2011; Newton *et al.*, 2011). Sorghum and millet is also very important as feed and fodder in sub-Saharan Africa. Table 4 shows the estimated 2005 and 2000 cattle population in each of the DCL priority farming systems. The size of the cattle population generally follows the size of human population, the number of poor and the area of crops ( Table 1, Table 4 and Table 7, respectively). Two farming systems stand out for their high population of cattle – *rainfed mixed* and *rice-wheat*, both in South Asia. However, high population and crop area in East Asia do not translate into the very high cattle populations seen in South Asia. For example, the three East Asia priority farming systems – *lowland rice*, *upland intensive mixed* and *temperate mixed* – have cattle populations in the middle of the range of the priority systems, although pig populations are larger. Other priority systems in the middle of the range include *cereal-root crop mixed*, *agro-pastoral millet sorghum*, *pastoral*, *extensive cereal-livestock* and *maize mixed*. While the *small-scale cereal livestock* system in Eastern Europe and Central Asia and the *dry rainfed* system in South Asia have relatively low cattle populations among the priority systems, livestock is clearly important in these systems.

**Table 4.** Cattle population (head 2005 and 2010).

FARMING SYSTEMS	REGION	Cattle Population (head, 2005)	Cattle Population (head, 2010)
**Cereal-root crop** **mixed**	SSA	41,036,700	31,938,300
**Maize mixed**	SSA	38,945,200	33,555,200
**Agro-pastoral millet/** **sorghum**	SSA	31,997,700	35,608,700
**Pastoral**	SSA	26,729,000	34,317,600
**Rice-wheat**	SA	91,835,504	77,835,904
**Rainfed mixed**	SA	95,861,104	80,452,200
**Dry rainfed**	SA	9,270,570	8,765,500
**Highland mixed**	MENA	6,716,780	6,727,600
**Rainfed mixed**	MENA	3,401,060	2,867,500
**Dryland mixed**	MENA	3,572,810	2,588,100
**Pastoral**	MENA	2,230,120	2,981,300
**Maize-beans** **(Mesoamerica)**	LAC	16,577,200	13,324,000
**Large scale cereal-** **vegetable**	EECA	16,938,000	8,206,600
**Small scale cereal-** **livestock**	EECA	5,228,780	4,028,700
**Extensive cereal-** **livestock**	EECA	25,352,900	12,425,700
**Lowland rice**	EAP	39,531,100	44,108,800
**Upland intensive** **mixed**	EAP	39,954,600	47,748,400
**Temperate mixed**	EAP	20,527,700	22,756,000

### Abiotic constraints

**Figure 2.**
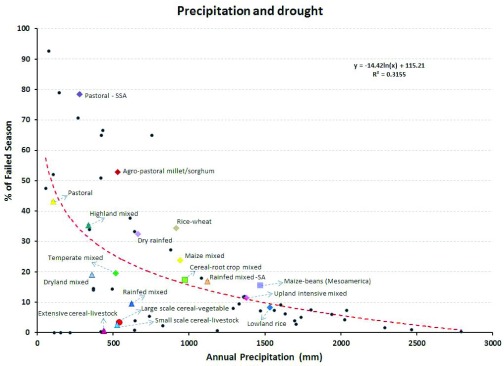
Average annual rainfall and average drought probability in 63 farming systems. Priority dryland cereal and legume farming systems are labeled.

The farming systems where dryland cereals and grain legumes are concentrated are particularly prone to drought and high temperatures ( Table 5). Farming systems in areas with relatively low (and variable) annual precipitation are more susceptible to failed growing seasons, as shown in Figure 2. These dryland systems, especially those with less than 1000 mm of annual precipitation, tend to have a higher probability of drought or a failed season, when precipitation does not meet crop requirements. Areas that have high probabilities of being affected by drought as shown by the potential drought impact index (PDII) include the *rainfed mixed* system in South Asia and the *agro-pastoral millet sorghum* and *pastoral* systems in sub-Saharan Africa ( Table 5). The *rice-wheat* system in South Asia also has a high PDII, where drought may particularly affect pearl millet and chickpea. Other systems that are particularly prone to drought include *cereal-root crop mixed* and *maize mixed* in sub-Saharan Africa and *dry rainfed* in South Asia.

**Table 5.** The farming systems where dryland cereals and grain legumes are concentrated are particularly prone to high temperatures and drought.

FARMING SYSTEMS	REGION	DLC Crop Area (ha)	Potential Drought Impact Index	Temperature Change 2050
**Cereal-root crop mixed**	SSA	21,327,541	2,971,040	2.48
**Maize mixed**	SSA	7,606,508	1,592,730	2.47
**Agro-pastoral millet/** **sorghum**	SSA	18,691,342	7,644,810	2.77
**Pastoral**	SSA	10,808,337	7,409,830	2.73
**Rice-wheat**	SA	11,282,838	4,431,820	2.83
**Rainfed mixed**	SA	30,763,078	7,556,180	2.48
**Dry rainfed**	SA	8,685,308	2,868,150	2.36
**Highland mixed**	MENA	2,961,344	98,050	3.01
**Rainfed mixed**	MENA	1,588,829	123,471	2.64
**Dryland mixed**	MENA	3,840,974	104,013	2.79
**Pastoral**	MENA	1,000,516	10,668	2.93
**Maize-beans** **(Mesoamerica)**	LAC	1,749,799	398,401	2.36
**Large scale cereal-** **vegetable**	EECA	6,947,991	86,502	2.82
**Small scale cereal-** **livestock**	EECA	2,550,258	1,849	2.82
**Extensive cereal-** **livestock**	EECA	9,160,822	17,198	3.31
**Lowland rice**	EAP	8,778,265	982,407	2.25
**Upland intensive** **mixed**	EAP	7,868,661	1,065,610	2.42
**Temperate mixed**	EAP	6,539,133	1,088,910	2.91

**Figure 3.**
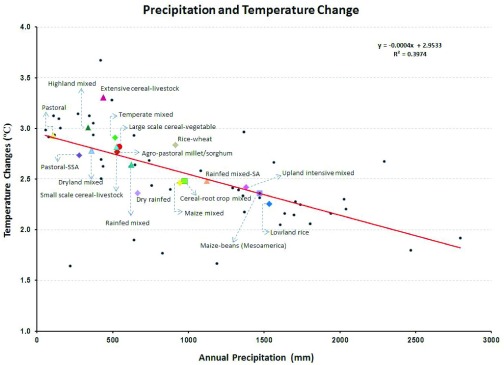
Average annual rainfall and average temperature change projected between the current temperature and 2050 temperature. Priority dryland cereal and legume farming systems are labeled.

The DCL crops are also expected to be constrained by the rising temperatures that come with climate change. There is a general tendency of the drier farming systems having higher expected temperature changes between now and 2050 ( Figure 3). Average temperature changes are expected to be between 2.4 and 3.4°C. The DCL priority farming systems in Eastern Europe and Central Asia could be particularly hard hit, with expected temperature rises of 3.3°C for *extensive cereal livestock* and 2.8°C for both *large-scale cereal vegetable* and *small-scale cereal livestock*. The estimated temperature change by 2050 in the *temperate mixed* system in East Asia is 2.9°C. For the *rice-wheat* system in South Asia and the *agro-pastoral millet sorghum* system in sub-Saharan Africa the expected change is 2.8°C, important expected changes because of their large area of DCL crop cultivation. The *rice-wheat* system has more than 11.2 million ha of DCL crops, while the *agro-pastoral millet sorghum* system has more than 18.6 million ha.

The soils of DCL priority farming systems present a number of abiotic constraints to DCL crop production. Table 6 shows some of the principal constraints identified by the DCL commodity programs as they affect priority farming systems ( DCL, 2015). The proportional area of farming systems with acid soil ranges from 9% in the *small-scale cereal livestock* system to 39% in the *rainfed mixed* system. The *cereal-root crop mixed* system is another one with a very large proportion – 37 percent – of its area exhibiting acid soils. Another system with a large area of acid soils is the *maize mixed* system in sub-Saharan Africa, with 27 percent of its area under this constraint. *Lowland rice* and *upland intensive mixed* in East Asia have nearly a quarter of their areas with acid soil constraints. These latter two systems also suffer from large areas with soils of low nutrient availability, with over one third of the area under this condition. Low nutrient availability is also an important constraint in the *cereal-root crop mixed*, *maize beans* and *rainfed mixed* systems, with proportional areas of 19, 14 and 10 percent of their total areas under this constraint, respectively. Salinity constraints are less problematic, with 13 of the 15 priority farming systems having less than 6% of their areas with this condition. The exceptions for soils with salinity constraints are the *rice-wheat* system in South Asia with 23 percent and the *temperate mixed* system in East Asia with 18 percent of their areas subject to salinity constraints. A group of farming systems has between 20 and 40 percent of their areas on soils with low moisture holding capacity – an important constraint in dryland systems due to the need for soils to store water for as long as possible. These systems include the *agro-pastoral millet sorghum* (38%), *pastoral* (30%) and *cereal-root crop mixed* (22%) farming systems in sub-Saharan Africa.

**Table 6.** The percentage area of each of the 18 priority farming system with soil constraints.

FARMING SYSTEMS	REGION	Acid Soil constraints (MEAN % of farming system)	Soil > 3 months dry season (MEAN % of farming system)	Soil subject to waterlogging (MEAN % of farming system)	Soil with low moisture holding capacity (MEAN % of farming system)	Soil with Low nutrient availability (MEAN % of farming system)	Soil with Salinity constraints (MEAN % of farming system)
**Cereal-root** **crop mixed**	SSA	37	1	14	22	19	1
**Maize mixed**	SSA	27	4	7	14	35	1
**Agro-pastoral** **millet/sorghum**	SSA	15	4	8	38	7	3
**Pastoral**	SSA	5	20	4	30	2	6
**Rice-wheat**	SA	19	21	7	5	4	23
**Rainfed mixed**	SA	39	2	3	12	10	2
**Dry rainfed**	SA	14	0	2	1	1	2
**Highland mixed**	MENA	2	36	2	2	0	5
**Rainfed mixed**	MENA	25	13	3	3	0	2
**Dryland mixed**	MENA	7	38	1	5	0	5
**Pastoral**	MENA	1	42	1	10	0	8
**Maize-beans** **(Mesoamerica)**	LAC	30	1	3	3	14	0
**Large scale cereal-** **vegetable**	EECA	17	5	11	6	0	5
**Small scale cereal-** **livestock**	EECA	9	31	2	1	1	2
**Extensive cereal-** **livestock**	EECA	11	2	15	6	0	1
**Lowland rice**	EAP	22	0	35	5	30	2
**Upland intensive** **mixed**	EAP	23	0	10	1	35	1
**Temperate mixed**	EAP	10	1	35	2	0	18

### Socioeconomics

The key DCL farming system regions are home to about half of the global population, including a massive number of people living in poverty ( Table 7). About 3.5 billion people live in these areas, 2.3 billion of them living in rural areas and 1.3 billion in towns and cities. The highest populations are in South Asia and East Asia. The *lowland rice* and *upland intensive mixed* systems in East Asia are two of the largest systems in terms of population, with roughly 851 and 501 million people in each respective system. Important South Asian farming systems include large numbers of urban and rural people – with over 400 million people in the *rainfed mixed* system and over 600 million people in the *rice-wheat* system. The remaining 14 DCL priority farming systems have a total of more than 960 million people.

**Table 7.** DCL farming system, population and poverty indicators. The key DCL farming systems are home to about one third of the global population, including an enormous number of people living in poverty.

FARMING SYSTEMS	REGION	2010 Population ('000)	2005 Population ('000)	2005 Rural Population ('000)	2005 Urban Population ('000)	Stunted Children ('000)	Stunting Prevalence	Poverty headcount (<$1/day) ('000)	Poverty headcount (<$2/day) ('000))	DCL Crop Area ('000 ha)
**Cereal-root crop** **mixed**	SSA	116,472	84,150	69,199	14,951	6,320	39	52,865	73,618	21,328
**Maize mixed**	SSA	125,279	96,684	72,837	23,847	6,314	41.1	51,310	68,988	7,607
**Agro-pastoral** **millet/sorghum**	SSA	70,806	54,864	37,892	16,972	3,133	37	30,899	40,999	18,691
**Pastoral**	SSA	51,662	39,705	29,677	10,027	3,228	35.5	13,369	20,871	10,808
**Rice-wheat**	SA	613,984	491,399	365,498	125,901	28,292	51.5	237,306	440,256	11,283
**Rainfed mixed**	SA	400,921	356,767	249,337	107,430	24,541	62.6	157,816	286,661	30,763
**Dry rainfed**	SA	47,017	45,600	33,544	12,056	3,610	65.5	18,074	32,620	8,685
**Highland mixed**	MENA	72,913	67,103	31,036	36,067	1,572	20.4	3,648	11,254	2,961
**Rainfed mixed**	MENA	47,798	38,815	13,852	24,963	499	16.3	1,666	6,415	1,589
**Dryland mixed**	MENA	56,966	47,224	18,093	29,132	750	18.7	1,128	4,380	3,841
**Pastoral**	MENA	38,441	33,845	16,798	17,047	1,668	21.9	988	4,444	1,001
**Maize-beans** **(Mesoamerica)**	LAC	88,137	76,106	28,686	47,420	2,838	35.9	4,684	9,278	1,750
**Large scale** **cereal-vegetable**	EECA	63,105	65,593	28,474	37,118	319	8.7	1,501	1,178	6,948
**Small scale** **cereal-livestock**	EECA	19,852	19,898	8,763	11,135	382	19.6	658	2,175	2,550
**Extensive** **cereal-livestock**	EECA	92,121	93,425	26,044	67,381	70	3.7	1,639	2,848	9,161
**Lowland rice**	EAP	851,260	785,701	496,073	289,627	13,360	31.8	117,021	264,030	8,778
**Upland** **intensive mixed**	EAP	501,857	502,323	358,539	143,783	15,427	33.6	84,484	193,653	7,869
**Temperate** **mixed**	EAP	285,014	260,574	138,989	121,585	2,594	21.6	36,416	82,927	6,539
**TOTAL**		**3,543,606**	**3,159,775**	**2,023,332**	**1,136,441**	**114,917**		**815,472**	**1,546,593**	**162,152**

The DCL priority farming systems are home to a large proportion of the world’s poor ( Table 7). According to year 2005 childhood stunting and $1 and $2 a day poverty indicators, about 60% of the world’s poor live within these 18 systems ( Table 7; FAO, 2007; Stanley Wood, personal communication). This large proportion is due to the importance of these systems in high-population countries like China and India, as well as farming systems spanning West and East Africa. Of the 63 global farming systems, the DCL priority systems include eight of the top 10 systems in terms of numbers of poor people. These eight DCL systems are *rice-wheat* and *rainfed mixed* in South Asia, *lowland rice*, *upland intensive mixed* and *temperate mixed* in East Asia and *cereal-root crop mixed*, *maize mixed* and *agro-pastoral millet sorghum* in sub-Saharan Africa.

Using the population of stunted children as a nutrition and poverty indicator, more than 60 percent of the 2005 global population of stunted children live within the DCL priority farming systems ( Table 7; de Onis *et al.*, 2012; FAO, 2007). Much of this poverty is concentrated in South Asia, East Asia and sub-Saharan Africa, regions with historically high rates of malnutrition. According to the stunting indicator, two farming systems stand out, both in South Asia. The *rice-wheat* and *rainfed mixed* systems have 28 and 24 million stunted children, respectively, with malnutrition levels exemplifying the high population density and well-known nutrition problems of these regions. In the *lowland rice* and *upland intensive mixed* systems of East Asia, the number of stunted children is about half of the South Asian systems mentioned previously, with 13 and 15 million stunted children respectively. In sub-Saharan Africa, the *maize mixed* and *agro-pastoral millet sorghum* systems have about six million stunted children each, half again as much as the East Asia systems mentioned above. Another seven farming systems across five world regions have between one and four million stunted children. The remaining five farming systems regions – two in the Middle East and North Africa regions and three in the Eastern Europe and Central Asia regions – have less than one million stunted children, mostly reflecting the lower overall populations of these regions.

### Where do DCL crops coincide?

**Figure 4.**
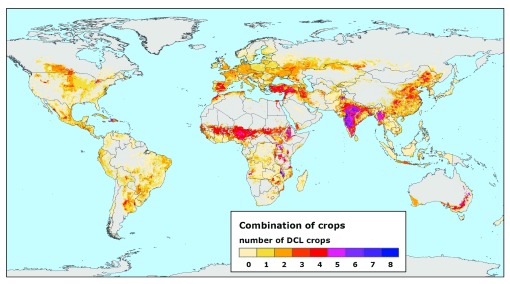
The DCL crops present a number of opportunities for bringing multiple technology options among different crops to the same geographic area. The map shows several core areas where 3 to 5 or more DCL crops are grown together.

**Figure 5.**
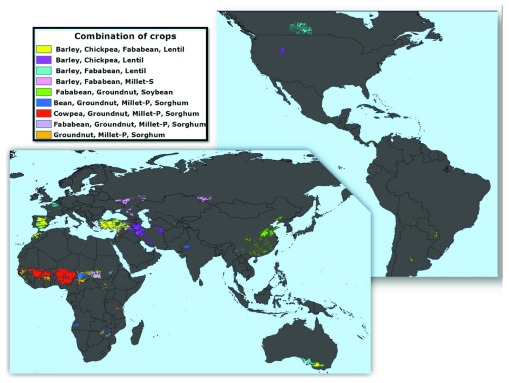
Crop combinations with the largest area.

The DCL crops present a number of opportunities for bringing multiple technology options among different crops to the same geographic area ( Figure 4). The map shows several core areas where three to five or more DCL crops are grown together. These core areas include (1) a large area spanning the Sahel region of West Africa, (2) a discontinuous cluster of areas in East Africa, (3) a large part of South Asia extending from India north to Pakistan and then east to Bangladesh and Myanmar, and (4) a large swath of area in the Middle East extending from Iran to Turkey. But there are also concentrations of multiple crops in Mexico and Central America, China and other regions. Figure 5 shows some of the crop combinations with the largest area. In the Sahel region, a huge area where groundnut, pearl millet and sorghum are grown together is found. The eastern part of this region contains systems that include these crops plus common bean, while the Western part of the region includes the same crops and much more cowpea cultivation. In the Middle East, the combination of barley, chickpea, lentil and faba bean, make up large cultivated areas within the region.

## Discussion and Conclusions

This study identified 18 farming systems globally that are important for dryland cereals and grain legumes agri-food systems. The most important of these systems, in terms of area and population, are found in South Asia and sub-Saharan Africa. The results discussed above suggests that these two regions deserve primary focus based on their relatively large cultivated area of DCL crops, large populations and high poverty. The farming systems in Latin America, the Middle East and North Africa, Central Asia and East Asia are also important. Research in any one region can have managed spillover effects in the others because the crops and biophysical conditions are similar across these regions. Interestingly, many DCL cereals and legumes show wide adaptability and have persisted in areas of moderate rainfall, such as the *maize mixed* farming system, and even in irrigated farming systems such as *lowland rice*. 

A focus on the 18 farming systems identified in this study in no way excludes any areas of the globe as areas where DCL should conduct and target research and development. It simply narrows down the DCL focus area to areas with substantial production of DCL commodities, with drylands and with substantial poverty and development problems. 

This result can be compared against two existing maps, both of which can be viewed on the website of the DCL Atlas. One map shows dryland ecologies and another shows the countries prioritized by the DCL research program ( DCL, 2015). The dryland ecologies map is solely based on dryness, as indicated by temperature, precipitation and evapotranspiration. Effectively, the map includes large areas where there are very few people and almost no cultivated land. The 18 farming systems identified in this research fall within the dryland ecologies map. Two partial exceptions to this pattern are the *maize-beans* system in Mesoamerica and the *rainfed mixed* system in India, where the boundaries of the farming system extend beyond the dryland ecology boundaries.

DCL’s target countries map – based on national-level data – was developed using a combination of factors, namely, target crop area, agricultural population, population under poverty, prevalence of child malnutrition, and to the extent that data was available, land degradation based on the satellite-derived Normalized Difference Vegetation Index (NDVI; Pettorelli *et al.*, 2005). The emphasis was on countries in dryland ecologies. In an effort to prioritize the large number of countries (51+), the focus was defined to be on sub-Saharan Africa and South Asia, where the area under the combined DCL was the highest among an assembled list of Low-Income Food-Deficit Countries ( Pingali & Stringer, 2003). The target countries map also agrees well with the map of 18 farming systems. However, one drawback of the target countries approach is that it cannot distinguish between data representing the crop distribution and agroecology of DCL crops on the one hand, and country level data that was used for priority setting on the other. The results of this study overcome that obstacle by combining farming systems and countries, and by taking a more detailed spatial approach at subnational pixel level, as opposed to country level.

The results of this study can also be compared to a previous study that used the same approach, but with 23 crops, including the major staples rice, wheat, cassava and maize, among others ( Hyman *et al.*, 2008). That study focused on developing-country agriculture and prioritized 15 farming systems, with an emphasis on regions with large cultivated crop areas and large numbers of people. Seven of this study’s farming systems were not included in the previous study – *pastoral*, *dry rainfed* (SA), *highland mixed* (MENA), *dryland mixed, large-scale cereal vegetable*, *small-scale cereal livestock* and *extensive cereal-livestock.* These seven systems are mostly focused on DCL crops, have generally lower populations and cultivated areas, have a greater tendency towards mixed livestock and cereal production and are found in areas with lower rainfall. Five of the 15 systems in the previous study do not appear in this study on DCL. Two of these systems are lowland and very wet – *rice* in South Asia and *root crop* in sub-Saharan Africa. The other three systems in the previous study but not found in this one are highland systems in South Asia, sub-Saharan Africa and East Asia. The eight systems found in both studies show the importance of DCL crops to the global agricultural research and development effort. Six of the most important farming systems globally from the previous study ( Hyman *et al.*, 2008) are also systems important for DCL. They are *rice-wheat* and *rainfed mixed* in South Asia, *cereal-root crop* and m *aize mixed* in sub-Saharan Africa and *upland intensive mixed* and *lowland rice* in East Asia. While the latter two systems have relatively small proportions of DCL crops, the absolute areas and benefiting populations are large in densely populated Southeast and East Asia.

Dryland cereal and legume crop distribution data show that South Asia and sub-Saharan Africa are the most important regions for crop improvement and adapted crop management practices. However, the proportional area of many DCL crops is often relatively low in regions where rice, wheat and maize are important staples. Nevertheless, the DCL crops are important in these regions for several reasons. Grain legumes in particular may be important as a rotation crop to support soil nitrogen fixation. Because livestock are important in many of the 18 farming system regions prioritized in this research, taking advantage of crop-livestock system synergies is an opportunity that should be explored, especially in relation to fodder. Also, the benefits of pasture and long term crop rotations in relation to soil improvement and reduction of plant disease can be considerable. The substantial ranges between yields in different regions of the world suggest considerable scope for closing yield gaps. These differences suggest substantial opportunities for increasing sub-Saharan Africa and South Asian yields.

Abiotic constraints are significant obstacles to improving DCL production. Previous research showed that farmers in these DCL priority farming systems face potential drought conditions that have a much higher risk compared to most other farming systems ( Hyman *et al.*, 2008). Rising temperatures in DCL farming systems will place a growing demand on farmers to cultivate heat tolerant crops, and to develop practices to protect these crops. Farming systems on the edge of the tropics or in the subtropics, as one moves away from the equator, are more likely to face rising temperatures with climate change. The combined effects of drought and heat in these farming systems pose a significant challenge. The areas in the 18 priority farming systems show considerable soil limitations. One of the most important is infertility, as indicated by soil acidity and low nutrient reserves – for which legume crops are valuable. Other important soil limitations are related to water. Long dry seasons limit the water availability in the soil, which is compounded in coarse-textured soils with low water holding capacity, for which modern crop management practices are applicable. The dryness of these systems also make them susceptible to salinity, another important soil constraint in the DCL priority systems.

Socioeconomic conditions in the DCL priority systems identified in this study indicate high levels of population and high poverty. There are both large rural and urban populations, suggesting potential positive supply and demand dynamics, especially so in sub-Saharan Africa and South Asia. These conditions suggest opportunities for developing market oriented production. Clearly much of the DCL crop production will continue to be derived from semi-subsistence agriculture. The high levels of malnutrition as indicated by childhood stunting, especially in South Asia and sub-Saharan Africa, can be addressed in part by nutritious DCL crops, which are often important sources of protein and micronutrients. Biofortification of DCL crops could be an important consideration in these areas. Clearly, the high rural and urban population found in, and depending on, DCL farming system regions suggest the importance of these systems for research and development aimed at improving agriculture and livelihoods.

The areas where DCL crops occur together present opportunities for improving the efficiency of research and development because fixed costs of research activities can be shared by different crop commodity programs. Testing the performance of crop varieties is typically carried out by national agricultural research institutes in collaboration with CGIAR centers. An integrated program to develop joint research could take advantage of different CGIAR centers or commodity programs carrying out research activities at common experiment stations of a national agricultural research institute. Two regions stand out where DCL crops occur together ( Figure 5). An initiative to work on multiple crops in the same sites may be attractive for (1) millet, sorghum, groundnut and cowpea crops in the Sahel region, and for (2) barley, chickpea, lentil and faba bean in the Middle East.

This study points out several areas for further agricultural research to improve productivity of dryland cereals and legumes. First, an effort can be made to update this analysis using more recent data with higher temporal resolution. Using recent data is particularly important for crop distribution and socioeconomic data. This type of analysis will surely benefit from higher spatial resolution of geospatial data in the future, a trend increasingly common with improving capacities to collect, store and process geographic information. Perhaps the most substantial gap in this study has been the lack of information on biotic constraints to crop production. Pests and diseases are often the most important threats facing farmers. But to date there are few consistent and standardized geographic assessments of the major pests and disease threats to crops, notwithstanding the progress made on wheat rust prevalence ( Singh *et al.*, 2008). Overcoming this obstacle would require a systematic effort to collect information on the occurrence of biotic constraints. A recent paper showed the potential of improving our knowledge of the geographic dimensions of agricultural biodiversity ( Castañeda *et al.*, 2016). Interestingly, that research showed that the dryland systems area of the Middle East and North Africa is a priority for collecting wild relatives of food crops. Our research suggested the importance of temperature and precipitation under climate change for the future of DCL crops. Research is needed on understanding the sensitivity of each crop to increases in temperature and to the duration of drought conditions. Research is also needed on understanding genotype by environment interactions for the DCL crops. Other staple crops such as maize, wheat and rice have a better track record in these types of studies, suggesting a higher potential return on investment for this type of research on DCL crops in the future. In relation to comprehensive analyses which position DCL crops in the full farming systems in the two priority regions for DCL crops, a new edited volume will be valuable, a forthcoming book titled *Farming Systems and Food Security in Africa: Priorities for Science and Policy Under Global Change*, edited by John Dixon, Dennis Garrity, Jean-Marc Boffa, Tim Williams, Tilahun Amede with Christopher Auricht, Rosemary Lott and George Mburathi. A similar comprehensive analysis is recommended for South Asia.

## Declarations

### Data availability

This research initiative developed two general types of data – digital spatial data of the world related to DCL crops and tabular data that summarizes the geographic information by farming system and country. All the spatial data used in the analysis can be accessed from the DCL Atlas at http://www.eatlasdcl.cgiar.org/. This online atlas includes data on the distribution of the 12 DCL crops, maps of predicted suitability of each crop, maps of abiotic constraints to crop production, maps of the biodiversity of relatives of each crop species, maps of socioeconomic conditions important for understanding the environment where these crops are grown and reference maps for putting all this information in the context.

Tabular data summarizing geographic information by farming system and country can be found in two dataset resources on the Dataverse repository:

*Harvard Dataverse*: Replication Data for: Priority regions for research on dryland cereals and legumes, 10.7910/DVN/EDMXSK ( Barona *et al.*, 2016a).

*Harvard Dataverse*: Characterization data on crops, production systems, abiotic constraints, population and poverty for farming system regions of the world, 10.7910/DVN/PLJ4SC ( Barona *et al.*, 2016b).
