# Supplementary material for: Priority regions for research on dryland cereals and legumes
Source: F1000Res. 2016 Jul 7;5:885. Originally published 2016 May 13. [Version 2] doi: 10.12688/f1000research.8657.2 (PMC4897757; doi:10.12688/f1000research.8657.2)
Supplement: Peer review history

# Peer review: Referee response for version 1

**DOI:** 10.5256/f1000research.9316.r14209

**Published:** 2016-06-06

**Authors:** Liangzhi You

**Affiliations:** 1 International Food Policy Research Institute, Washington, DC, USA

Efficient allocation of limited research resources is a central policy issue for international development agencies, developing-country governments, and their agricultural research institutes. Priority setting in agricultural research systems is a principal means of ensuring the effectiveness of investment in agricultural research. The new CGIAR has emphasize more on research impact on development. As part of the CGIAR research program on dryland cereal and legumes, this paper developed an open access dataset and information resource that provides the basis for future analysis of the geographic dimensions of dryland cereals and legumes.

 The foremost important contribution of this paper is to integrate multiple global spatial databases on cropping system, livestock and biophysical conditions for setting the priority. These newly available datasets allows the priority setting at crop-type level in a truly spatially explicit manner. The use of geographic information system (GIS) and spatial analysis captured the effects of agroecological variation and huge spatial heterogeneity  of farming system, the response of agricultural technologies, the technological spillovers.

 The next strength of the paper is its open access. The paper itself is open access. The datasets used in the paper is also openly available. Interested users could download these datasets and repeat/improved the analysis undertook in the paper.

 The weakness is the methodology section. While the paper spent quite some effort/sections on data sources, summary of results, the methodology is quite short and not clear. Admittedly the readers are referred to the well-known farming system framework by Dixon et al., and the authors’ previous work. The methodology should be updated and improved with the new datasets and tools. A brief summary is warranted. Technical details on how to deal with spatial fragmentation (e.g due to many different data layers) would be helpful for those who would like to learn and repeat the analysis of this paper.

I have read this submission. I believe that I have an appropriate level of expertise to confirm that it is of an acceptable scientific standard.

# Peer review: Referee response for version 1

**DOI:** 10.5256/f1000research.9316.r13828

**Published:** 2016-05-25

**Authors:** Jeffrey D. Ehlers

**Affiliations:** 1 Bill and Melinda Gates Foundation, Seattle, WA, USA

Positives:

 Using a basic farming system characterization schema developed some 15 years prior, this paper selects 18 of these where key dryland cereals and legume crops (DCL crops) are important, and refines their boundaries using new data and GIS resources. This work then provides a needed update to the earlier 2001 work. Each of these cropping system units is then characterized with DCL crop information (area grown, productivity, proportion of area, present and future drought severity, as well as socio-economic data such as population and poverty, as well as with cattle population data, to create an information rich view of these systems. This work therefore helps in discovering appropriate and high leverage investment opportunities that support economic development. Another value of the article is that it serves to publicize an open access dataset and information resources that can be used in future analysis of DCL systems.

 A valuable contribution of this work is the integration of datasets/information across the DCL crop, agro-ecological and socio-economic dimensions, especially at sub-national level. At the same time there is little doubt the data sets used in the analysis were of highly variable quality. It seems like it would be good to face this head-on and include more discussion around data quality issues and how much confidence the reader should put in the analysis given that some of the data likely to be of low to moderate quality.  How are conclusions affected when integrating datasets on one topic that are of high quality with another for another parameter that are of poor quality?

 The authors usefully point out some important research areas that need attention in the DCL crops to make the current maps more actionable. These include things like relative response to higher temperature, genotype by environment interactions but also that ‘one of the most important shortcomings in this type of analysis has been the lack of data with which to produce a biotic constraints map layer as these types of constraints can be key drivers of crop distribution.

I have read this submission. I believe that I have an appropriate level of expertise to confirm that it is of an acceptable scientific standard.
